# Dilated Cardiomyopathy Mutation (R134W) in Mouse Cardiac Troponin T Induces Greater Contractile Deficits against α-Myosin Heavy Chain than against β-Myosin Heavy Chain

**DOI:** 10.3389/fphys.2016.00443

**Published:** 2016-10-04

**Authors:** Sampath K. Gollapudi, Murali Chandra

**Affiliations:** Department of Integrative Physiology and Neuroscience, Washington State UniversityPullman, WA, USA

**Keywords:** dilated cardiomyopathy, Troponin T, myosin heavy chain, contractile dynamics, thin filament function, myofilament cooperativity

## Abstract

Many studies have demonstrated that depressed myofilament Ca^2+^ sensitivity is common to dilated cardiomyopathy (DCM) in humans. However, it remains unclear whether a single determinant—such as myofilament Ca^2+^ sensitivity—is sufficient to characterize all cases of DCM because the severity of disease varies widely with a given mutation. Because dynamic features dominate in the heart muscle, alterations in dynamic contractile parameters may offer better insight on the molecular mechanisms that underlie disparate effects of DCM mutations on cardiac phenotypes. Dynamic features are dominated by myofilament cooperativity that stem from different sources. One such source is the strong tropomyosin binding region in troponin T (TnT), which is known to modulate crossbridge (XB) recruitment dynamics in a myosin heavy chain (MHC)-dependent manner. Therefore, we hypothesized that the effects of DCM-linked mutations in TnT on contractile dynamics would be differently modulated by α- and β-MHC. After reconstitution with the mouse TnT equivalent (TnT_R134W_) of the human DCM mutation (R131W), we measured dynamic contractile parameters in detergent-skinned cardiac muscle fiber bundles from normal (α-MHC) and transgenic mice (β-MHC). TnT_R134W_ significantly attenuated the rate constants of tension redevelopment, XB recruitment dynamics, XB distortion dynamics, and the magnitude of length-mediated XB recruitment only in α-MHC fiber bundles. TnT_R134W_ decreased myofilament Ca^2+^ sensitivity to a greater extent in α-MHC (0.14 pCa units) than in β-MHC fiber bundles (0.08 pCa units). Thus, our data demonstrate that TnT_R134W_ induces a more severe DCM-like contractile phenotype against α-MHC than against β-MHC background.

## Introduction

Dilated Cardiomyopathy (DCM), a disease caused by mutations in many sarcomeric proteins, is characterized by systolic dysfunction and ventricular dilatation (Kushner et al., 2006; Hershberger et al., 2009; Willott et al., 2010; Marston, 2011; Lu et al., 2013). *In vitro* studies of DCM-causing mutations in cardiac Troponin T (TnT) generally correlate depressed myofilament Ca^2+^ sensitivity to systolic dysfunction (Kushner et al., 2006; Hershberger et al., 2009), with some exceptions (Mirza et al., 2005). Therefore, it remains unclear whether a single determinant, such as myofilament Ca^2+^ sensitivity, is sufficient to characterize all cases of DCM because the severity of disease varies widely with a given mutation. Proper pumping actions of the heart dictates that—in addition to normal Ca^2+^ dynamics—both the magnitude and speed of contraction are not only sustained but adjusted properly on a beat-to-beat basis. Because contractile dynamics are strongly dependent on thin filament cooperativity, and such cooperativity is modulated by the central region (CR) of TnT (Schaertl et al., 1995; Tobacman et al., 2002; Gollapudi et al., 2013), mutations in the CR of TnT are expected to affect myofilament activation by modifying dynamic features of cardiac contractile activation. A better assessment of disparate cardiac phenotypes is made possible when studies account for the mutation-mediated effect on dynamic contractile function because dynamic aspects dominate heart function under physiological conditions.

The focus of this study is the DCM-related mutation, R131W (Mogensen et al., 2004), which lies within the CR (residues 80–180) of human TnT. We previously demonstrated that the CR of TnT plays an important role in tuning the dynamics of crossbridge (XB) recruitment in cardiac muscle by modulating cooperative mechanisms within thin filaments (Gollapudi et al., 2013). Such actions likely involve strong CR-Tropomyosin (Tm) interactions that take place near the overlap junction of adjacent Tm dimers (Jackson et al., 1975; Pearlstone and Smillie, 1977; Palm et al., 2001; Hinkle and Tobacman, 2003; Gollapudi et al., 2013). Therefore, the R131W mutation in TnT may perturb CR-Tm interactions to modulate XB recruitment dynamics. Given that the TnT-mediated function is dependent on the myosin heavy chain (MHC) isoform (Ford et al., 2012; Chandra et al., 2015; Gollapudi et al., 2015; Gollapudi and Chandra, 2016), we hypothesized that the effects of DCM-linked mutations in TnT on contractile dynamics would be differently modulated by α- and β-MHC. To better understand the molecular mechanisms that lead to contractile dysfunction, especially in relevance to humans, it is important to consider the differential impact of α- and β-MHC isoforms on contractile dynamics. This is because previous studies have demonstrated that the effects of cardiomyopathy mutations in TnT on steady-state and/or dynamic contractile features are differently modulated by α- and β-MHC isoforms (Ford et al., 2012; Chandra et al., 2015; Gollapudi et al., 2015; Gollapudi and Chandra, 2016).

To test our hypothesis, we generated a recombinant mouse TnT equivalent (TnT_R134W_) of the human DCM mutation, R131W. Various indices of steady-state and dynamic contractile function were measured in normal (α-MHC) and transgenic mouse (β-MHC) cardiac muscle fiber bundles reconstituted with wild-type (WT) TnT (TnT_WT_) or TnT_R134W_. Dynamic contractile features mediated by TnT_R134W_ were altered only in α-MHC fiber bundles, despite desensitization of myofilaments to Ca^2+^ to a different degree in α- and β-MHC fiber bundles. For instance, TnT_R134W_ attenuated rate constants of tension redevelopment, XB recruitment dynamics, XB distortion dynamics and the magnitude of length-mediated XB recruitment only in α-MHC fiber bundles. We will discuss the correlation between altered contractile dynamics and a more severe DCM-like contractile phenotype against α-MHC than against β-MHC background.

## Materials and methods

### Animal treatment protocols

3–4 month-old male mice were used in this study. WT C57BL/6N-strain (non-TG, NTG) mice were acquired from Simonsen's laboratories (Gilroy, CA). β-MHC TG mice were a generous gift from Dr. Jil Tardiff, University of Arizona, Tucson, AZ. The generation and characterization of β-MHC TG mice was as previously described (Krenz et al., 2003, 2007; Krenz and Robbins, 2004; Gollapudi et al., 2015). Mice were carefully handled to minimize pain and suffering, as per the established guidelines of the National Academy of Sciences *Guide for the Care and Use of Laboratory Animals*. All procedures used for the treatment of mice were approved by the board of Washington State University Institutional Animal Care and Use Committee.

### Purification of recombinant mouse cardiac Tn subunits

*c-myc* tagged mouse TnT_WT_ and TnT_R134W_ genes were synthesized (GenScript USA Inc., Piscataway, NJ) after codon optimization for enhanced protein expression. *c-myc* tagged TnT_WT_ served as the control in our study. Recombinant mouse TnT (TnT_WT_ and TnT_R134W_), mouse TnI, and mouse TnC were generated and purified, as described previously (Guo et al., 1994; Pan and Johnson, 1996; Chandra et al., 1999; Gollapudi and Chandra, 2012; Mamidi et al., 2013). Recombinant proteins were cloned into the T7 promoter-based pSBETa vector and expressed in BL21^*^DE3 cells (Novagen, Madison, WI) for protein synthesis. All proteins were purified using ion-exchange chromatography techniques. TnT_WT_ and TnT_R134W_ were purified by anion-exchange chromatography on a DEAE fast Sepharose column (Chandra et al., 1999; Gollapudi and Chandra, 2012; Mamidi et al., 2013; Gollapudi et al., 2015), TnI was purified by cation-exchange chromatography on a CM Sepharose column (Guo et al., 1994; Gollapudi and Chandra, 2012; Mamidi et al., 2013; Gollapudi et al., 2015), and TnC was purified by anion-exchange chromatography on a DE-52 column (Pan and Johnson, 1996; Gollapudi and Chandra, 2012; Mamidi et al., 2013; Gollapudi et al., 2015). All eluted fractions containing pure proteins were pooled and dialyzed extensively against deionized water containing 15 mM β-mercaptoethanol, lyophilized, and stored at −80°C for long-term use.

### Preparation of detergent-skinned cardiac muscle fiber bundles and Tn reconstitution

Left ventricular papillary muscle bundles were isolated from deeply-anesthetized (Isoflurane) mice, and further dissected into smaller muscle fiber bundles (~0.15 mm in cross-section and 2.0–2.5 mm in length) in high-relaxing (HR) solution (Chandra et al., 2006, 2007; Gollapudi et al., 2015). The HR solution contained 20 mM 2,3-butanedione monoxime (BDM), 50 mM N,N-bis (2-hydroxyethyl)-2-amino-ethane-sulfonic acid (BES), 20 mM ethylene glycol tetra-acetic acid (EGTA), 6.29 mM magnesium chloride (MgCl_2_), 6.09 mM disodium hydrate salt of adenosine triphosphate (Na_2_ATP), 30.83 mM potassium propionate (K-Prop), 10 mM sodium azide (NaN_3_), 1.0 mM dithiothreitol (DTT), and 4 mM benzamidine hydrochloric acid (Benz HCl). Fresh protease inhibitors [in μM: 5 bestatin, 2 E-64, 10 leupeptin, 1 pepstatin, and 200 phenylmethylsulfonyl fluoride (PMSF)] were also included in the HR solution. The pH of the HR solution was adjusted to 7.0 and the ionic strength to 180 mM. The smaller muscle fiber bundles were detergent-skinned overnight at 4°C in HR solution containing 1% Triton X-100.

Recombinant Tn subunits were reconstituted into detergent-skinned muscle fiber bundles, as described elsewhere (Chandra et al., 2006, 2007; Mamidi et al., 2013; Gollapudi et al., 2015). Briefly, TnT_WT_ or TnT_R134W_ (0.9 mg/ml, W/V) and TnI (1.0 mg/ml, W/V) were solubilized in an extraction buffer (*buffer 1*) containing the following (in M): 0.05 Tris-HCl (pH 8.0), 6.0 Urea, 1.0 KCl. High salt and urea in the extraction buffer were removed by successive dialysis against *buffers 2-4*, whose compositions are listed below.

*Buffer 2* (in M): 0.050 Tris-HCl, 4 urea, 0.7 KCl (pH 8.0 at 4°C)
*Buffer 3* (in M): 0.050 Tris-HCl, 2 urea, 0.5 KCl (pH 8.0 at 4°C)
*Buffer 4* (in mM): 50 BES, 200 KCl, 10 BDM, 6.27 MgCl_2_, 5 EGTA (pH 7.0 at 20°C)

All *buffers* (*1-4*) included several protease inhibitors (0.2 mM PMSF, 2 mM Benz HCl, 1 mM DTT, and 0.01% NaN_3_). Any undissolved protein in the extraction buffer was removed by spinning it at 3000 rpm for 15 min. Detergent-skinned fiber bundles were treated with the extraction buffer containing TnT_WT_+TnI or TnT_R134W_+TnI for ~3 h at room temperature (22°C) with gentle stirring. Muscle fiber bundles were then washed twice (10 min each) using *buffer 4* and incubated overnight at 4°C in HR solution containing TnC (3.0 mg/ml, W/V).

### Western blot analysis

Reconstituted muscle fiber bundles were solubilized in a muscle protein extraction buffer containing the following: 2.5% SDS, 10% glycerol, 50 mM tris base (pH 6.8 at 4°C), 1 mM DTT, 1 mM PMSF, 4 mM Benz HCl, and a fresh cocktail of phosphatase (PhosSTOP) and protease inhibitors (E 64, Leupeptin, and Bestatin). The final concentration of all solubilized protein samples was adjusted to 2 mg/ml using the protein loading dye (125 mM Tris-HCl (pH 6.8), 20% glycerol, 2% SDS, 0.01% bromophenol blue, and 50 mM β-mercaptoethanol). 5 μg of each protein sample was loaded and run on an 8% SDS-gel for optimal separation of *c-myc* tagged recombinant and endogenous TnT (Gollapudi et al., 2012, 2013; Mamidi et al., 2013). Proteins were then transferred to a polyvinylidene difluoride membrane and TnT was probed using a monoclonal anti-TnT primary antibody (M401134, Fitzgerald Industries Int, Concord, MA), followed by HRP-labeled anti-mouse secondary antibody (RPN 2132, Amersham Biosciences, Piscataway, NJ). The percentage incorporation of the exogenous Tn was determined by the densitometric analysis of the TnT band profiles on the Western blot using ImageJ software (acquired from NIH at http://rsbweb.nih.gov/ij/).

### PCA solutions and their compositions

For tension measurements, muscle fiber bundles were exposed to various solutions with pCa (= −log of [Ca^2+^]_free_) ranging from 9.0 to 4.3. The compositions of pCa 9.0 and 4.3 solutions were based on the program by Fabiato and Fabiato (1979), and are listed below.

*pCa 9.0 (in mM)*: 50 BES, 5 NaN_3_, 10 phosphoenol pyruvate (PEP), 10 EGTA, 0.024 calcium chloride (CaCl_2_), 6.87 MgCl_2_, 5.83 Na_2_ATP, and 51.14 K-Prop
*pCa 4.3 (in mM)*: 50 BES, 5 NaN_3_, 10 PEP, 10 EGTA, 10.11 CaCl_2_, 6.61 MgCl_2_, 5.95 Na_2_ATP, and 31 K-Prop

In addition, the pCa 9.0 and 4.3 solutions contained 0.5 mg/ml pyruvate kinase, 0.05 mg/ml lactate dehydrogenase, along with fresh protease inhibitors [(in μM): 10 leupeptin, 1000 pepstatin, 100 PMSF, 20 diadenosine penta-phosphate, 10 oligomycin]. The pH and ionic strength of pCa 9.0 and 4.3 solutions were adjusted to 7.0 and 180 mM, respectively.

### Measurements of steady-state isometric tension and ATPase activity

Simultaneous measurements of steady-state isometric tension and ATPase activity were made, as described previously (de Tombe and Stienen, 1995; Stienen et al., 1995; Chandra et al., 2006, 2007; Gollapudi et al., 2015). T-shaped aluminum clips were used to attach muscle fiber bundles between a motor arm (322C, Aurora Scientific Inc., Ontario, Canada) and a force transducer (AE 801, Sensor One Technologies Corp., Sausalito, CA). The sarcomere length (SL) of the muscle fiber bundles was set to 2.3 μm in HR solution by laser diffraction. Each fiber bundle was subjected to two cycles of maximal activation (pCa 4.3) and relaxation (pCa 9.0), and the SL was re-adjusted to 2.3 μm if necessary. The cross-sectional area (CSA) and the initial muscle length (ML) corresponding to the SL of 2.3 μm were measured for each preparation. Muscle fiber bundles were then bathed in various solutions with pCa ranging from 9.0 to 4.3, one at a time, in a constantly-stirred chamber. The fiber-elicited responses in steady-state force and ATPase activity were recorded on a computer at a sampling frequency of 1 kHz. All measurements were made at 20°C.

Measurements of steady-state ATPase activity under isometric conditions were based on an enzymatically coupled assay, as described previously (de Tombe and Stienen, 1995; Stienen et al., 1995; Chandra et al., 2006, 2007; Gollapudi et al., 2015). Tension cost was determined as the slope of the linear relationship between steady-state tension and ATPase activity at various pCa (de Tombe and Stienen, 1995; Stienen et al., 1995).

### Mechano-dynamic studies

**Figure 1:**
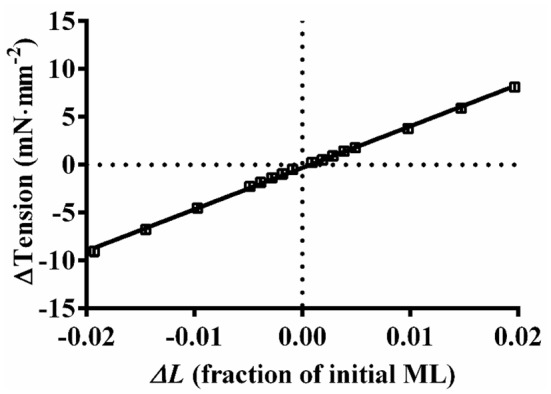
**Relationship between changes in the steady-state force responses and the imposed muscle length changes**. Five individual mouse fiber bundles (α-MHC) were subjected to various amplitude stretch/release perturbations (Δ*L*; ±0.1%, ±0.2%, ±0.3%, ±0.4%, ±0.5%, ±1.0%, ±1.5%, and ±2.0% of ML) and the corresponding steady-state force responses (Δ*T*) were recorded. The trace connecting the data (squares) represents the linear regression fit for the averaged Δ*T*-Δ*L* relationship from five muscle fiber bundles. The *r*^2^ value of the linear regression fit was 0.998, clearly indicating that the force scaled linearly with ML. Data are presented as mean ± SE. Standard error bars are smaller than symbols.

Fully activated muscle fiber bundles were subjected to various amplitude stretch/release perturbations. First, we wanted to test if force scaled linearly with muscle length changes; therefore, we subjected the muscle fiber bundles to varying amplitudes of length changes (±0.1% to ±2.0%). Experimental data showing the averaged relationship between force changes (Δ*T*) and ML changes (Δ*L*) in Figure 1 clearly indicates that force scales linearly with ML. We used the previously established protocol (Ford et al., 2010) to record force responses to varying amplitude length changes (±0.5, ±1.0, ±1.5, and ±2.0% of ML). Force and length data were sampled at 2 kHz. A nonlinear recruitment-distortion (NLRD) model was fitted to this family of force responses to estimate the following four model parameters: the magnitude of instantaneous increase in stiffness caused by a sudden increase in ML (*E*_D_); the rate by which the sudden ML-induced increase in stiffness decays to a minimum (*c*); the rate by which a new steady-state force is attained due to the recruitment of new force-bearing XBs, following an increase in ML (*b*); and the magnitude of increase in the steady-state stiffness caused by the ML-mediated increase in the number of newly-recruited force-bearing XBs (*E*_*R*_). More details on step perturbation protocol and the physiological significance of NLRD model parameters are provided in our previously published works (Ford et al., 2010; Gollapudi et al., 2012; Chandra et al., 2015).

### Measurement of rate of tension redevelopment (*k*_tr_)

The measurement of *k*_tr_ was based upon the force response to a slightly modified version of the large slack-restretch ML maneuver, originally designed by Brenner and Eisenberg (1986). In brief, the muscle fiber in the steady-state of maximal Ca^2+^ activation (pCa 4.3) was first subjected to a rapid (1 ms) release by 10% of its ML using a high speed length-control device (322C, Aurora Scientific Inc., Ontario, Canada). After holding the fiber at the decreased length for 25 ms, it is quickly stretched past its ML by 10%, following which it was rapidly brought back to its ML and allowed to redevelop force. *k*_tr_ was estimated by fitting the following mono-exponential function to the rising phase of the resulting force (*F*) response:

$$\begin{array}{l} F(t)\;=\;(F_{\text{ss}}\;-F_{\text{res}})(1\;-\;{\text{e}}^{-k_{\text{tr}}t})\;+\;{\text{F}}_{\text{res}} \end{array}$$

where *F*_ss_ is steady-state force and *F*_res_ is residual force.

### Data analysis

Normalized pCa-tension relationships were fitted to the Hill equation to derive pCa_50_ (an index of myofilament Ca^2+^ sensitivity) and *n*_H_ (an index of myofilament cooperativity). We used a two-way ANOVA to analyze the contractile function parameters because our experimental model involved two factors, TnT (TnT_WT_ and TnT_R134W_) and MHC (α-MHC and β-MHC). First, we assessed if the MHC-TnT interaction effect on a given contractile parameter was significant. A significant MHC-TnT interaction effect does not suggest a direct interaction between MHC and TnT but it demonstrates that the effects of TnT_R134W_ on a parameter are dissimilar in α- and β-MHC fiber bundles. When the MHC-TnT interaction effect was not significant, we interpreted the main effect of TnT. To probe the cause for a significant MHC-TnT interaction effect or a main effect of TnT, multiple *post-hoc t*-tests were carried out using uncorrected Fisher's Least Significant Difference (LSD) method. Statistical significance was set at *P* < 0.05. Data are expressed as mean ± standard error of the mean (SEM).

## Results

### Incorporation levels of recombinant TnT in α- and β-MHC fiber bundles

**Figure 2:**
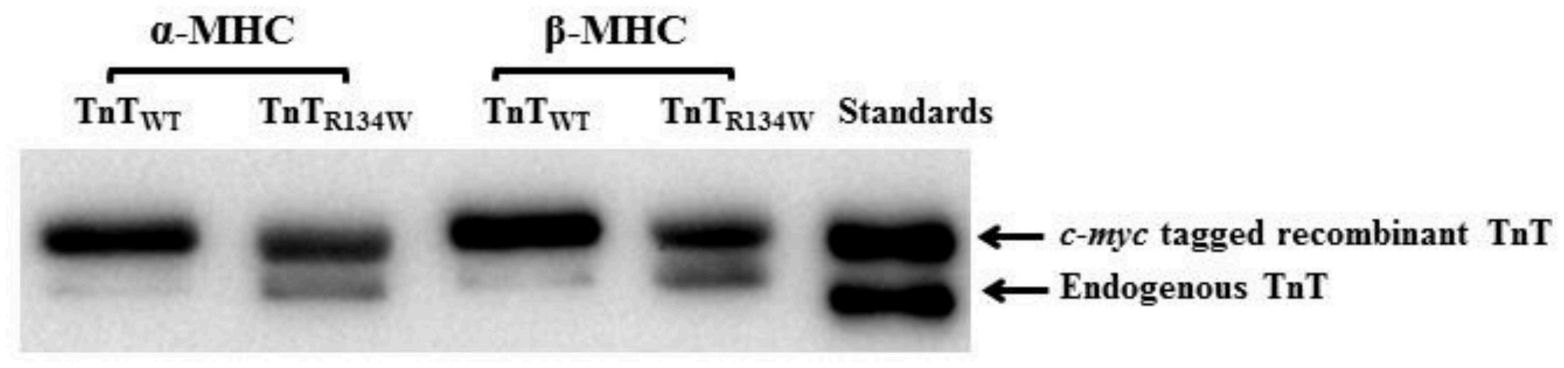
**Western blot showing the incorporation levels of recombinant TnT in α- and β-MHC fiber bundles**. Reconstituted fiber bundles were solubilized in 2.5% SDS solution and their final concentrations were adjusted to 2 mg/ml. Equal amounts (5 μg) of standardized protein samples were loaded and run on an 8% small SDS-gel for optimal separation of recombinant and endogenous TnT. Proteins were then transferred onto a PVDF membrane and TnT was probed using an anti-TnT primary, followed by an anti-TnT secondary antibody.

We have previously demonstrated that the expression level of β-MHC in TG mouse hearts was ~70% of the total MHC (Gollapudi et al., 2015). This overexpression of β-MHC had no impact on either the stoichiometry of other sarcomeric proteins or the phosphorylation levels of contractile regulatory proteins (Gollapudi et al., 2015). We used the Western blot to quantify the extent of recombinant Tn incorporation into muscle fiber bundles. The addition of an 11-amino acid *c-myc* tag at the N-terminus of recombinant TnT proteins (TnT_WT_ or TnT_R134W_) allowed us to separate the recombinant and endogenous TnT on an SDS gel, and to assess the extent of recombinant Tn incorporation in muscle fiber bundles. The inclusion of *c*-*myc* epitope had no impact on the TnT-mediated function in cardiac muscle (Tardiff et al., 1998; Montgomery et al., 2001). A representative Western blot showing the incorporation levels of recombinant TnT in α- and β-MHC fiber bundles is presented in Figure 2. Densitometric analysis revealed that the incorporation levels of TnT_WT_ and TnT_R134W_ in α-MHC fiber bundles were 93% and 74%, while those in β-MHC fiber bundles were 90% and 72%, respectively. Similar incorporation levels of TnT_R134W_ in both α- and β-MHC fiber bundles provided a good model to probe the interplay between TnT_R134W_- and MHC-mediated effects on various contractile parameters.

### TnT_R134W_-mediated impact on Ca^2+^-activated maximal tension and *E*_D_ in α- and β-MHC fiber bundles

We assessed whether TnT_R134W_ altered maximal activation in an MHC-dependent manner by analyzing the steady-state tension measurements at pCa 4.3. Two-way ANOVA of maximal tension did not reveal a significant MHC-TnT interaction effect (*P* = 0.35) or a main effect of TnT (*P* = 0.50). This is because TnT_R134W_ showed no impact on maximal tension in either α- or β-MHC fiber bundles. The mean ± SEM values of maximal tension (in mN·mm^−2^) in α-MHC+TnT_WT_ and α-MHC+TnT_R134W_ fiber bundles were 46.82 ± 1.03 (*n* = 13) and 46.11 ± 1.60 (*n* = 12), while those in β-MHC+TnT_WT_ and β-MHC+TnT_R134W_ fiber bundles were 45.50 ± 1.16 (*n* = 14) and 49.58 ± 1.24 (*n* = 14), respectively.

Previously, we have demonstrated that maximal tension is correlated to *E*_D_ (Campbell et al., 2004; Mamidi et al., 2013; Chandra et al., 2015). Therefore, to support our observations in maximal tension, we assessed *E*_D_. *E*_D_ is an approximate measure of the number of force-bearing XBs in the isometric steady-state prior to ML change (Campbell et al., 2004; Ford et al., 2010). Two-way ANOVA did not show a significant MHC-TnT interaction effect (*P* = 0.37) on *E*_D_ or a main effect of TnT (*P* = 0.14). Thus, TnT_R134W_ did not alter *E*_D_ in either α- or β-MHC fiber bundles. The mean ± SEM values of *E*_D_ (in mN·mm^−3^) in α-MHC+TnT_WT_ and α-MHC+TnT_R134W_ fiber bundles were 1041 ± 42 (*n* = 13) and 895 ± 49 (*n* = 12), while those in β-MHC+TnT_WT_ and β-MHC+TnT_R134W_ fiber bundles were 980 ± 59 (*n* = 14) and 951 ± 29 (*n* = 14), respectively. Similar observations in both maximal tension and *E*_D_ substantiate that TnT_R134W_ did not affect maximal activation regardless of the MHC isoform.

### TnT_R134W_-mediated impact on the pCa-tension relationship in α- and β-MHC fiber bundles

**Figure 3:**
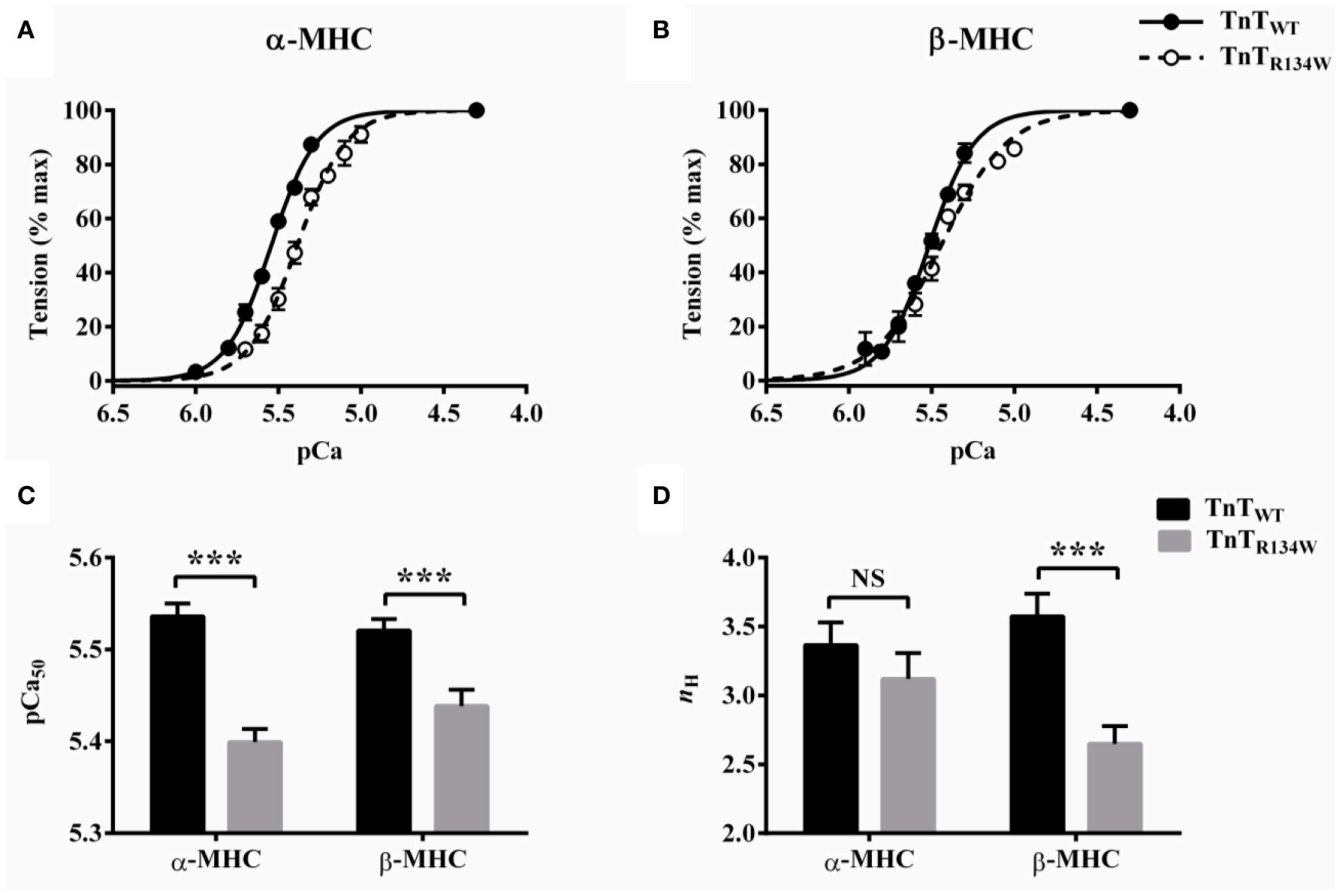
**Effect of TnT_**R134W**_ on pCa-tension relationships in α- and β-MHC fiber bundles**. Steady-state tensions, normalized to the value at pCa 4.3, were plotted against pCa to construct the pCa-tension relationship. Hill model was fitted to these pCa-tension relationships to estimate pCa_50_ (myofilament Ca^2+^ sensitivity) and *n*_H_ (myofilament cooperativity). TnT_R134W_-mediated impact on pCa-tension relationship in **(A)** α-MHC and **(B)** β-MHC fiber bundles. Traces connecting the data (empty or filled circles) are the Hill model fits. TnT_R134W_-mediated impact on **(C)** pCa_50_ and **(D)** *n*_H_ in α- and β-MHC fiber bundles. Statistical differences were analyzed by two-way ANOVA and subsequent *post-hoc t*-tests using Fishers LSD method. ^***^P < 0.001 indicate a significant result compared to TnT_WT_ (NS, not significant). The number of fiber bundles measured is as follows: 13 for α-MHC+TnT_WT_, 12 for α-MHC+TnT_R134W_, 14 for β-MHC+TnT_WT_, and 14 for β-MHC+TnT_R134W_. Data are presented as mean ± SE. Standard error bars are smaller than symbols in some cases.

A comparison of pCa-tension relationships showed that TnT_R134W_ induced a larger rightward shift in the pCa-tension relationship in α-MHC fiber bundles (Figure 3A) than in β-MHC fiber bundles (Figure 3B). A closer examination of the pCa-tension relationships also revealed that TnT_R134W_ did not alter the steepness of the pCa-tension relationship in α-MHC fiber bundles (Figure 3A) but it decreased the steepness in β-MHC fiber bundles (Figure 3B). To quantify the magnitude of such effects in α- and β-MHC fiber bundles, we analyzed the Hill model-derived parameters, pCa_50_ (myofilament Ca^2+^ sensitivity) and *n*_H_ (myofilament cooperativity). Two-way ANOVA of pCa_50_ did not show a significant MHC-TnT interaction effect (*P* = 0.17) but showed a significant main effect of TnT (*P* < 0.001). *Post-hoc* analysis revealed that TnT_R134W_ significantly decreased pCa_50_ in both α- and β-MHC fiber bundles; however, the magnitude of attenuation was different (Figure 3C). For example, TnT_R134W_ significantly attenuated pCa_50_ by 0.14 pCa units (*P* < 0.001) in α-MHC fiber bundles and by 0.08 pCa units (*P* < 0.001) in β-MHC fiber bundles. These observations suggest that TnT_R134W_ decreases myofilament Ca^2+^ sensitivity to a greater extent in the presence of α-MHC than in the presence of β-MHC. To quantify such changes in pCa_50_ in terms of tension, we also compared the steady-state tension data at submaximal Ca^2+^ activation (pCa 5.5) among groups. Our analysis showed that TnT_R134W_ significantly attenuated tension at pCa 5.5 by 46% (*P* < 0.001) in α-MHC fiber bundles and by 26% (*P* < 0.01) in β-MHC fiber bundles. These observations substantiate that, at submaximal Ca^2+^ levels, the attenuating effect of TnT_R134W_ on thin filament activation is stronger in α-MHC than in β-MHC fiber bundles.

Two-way ANOVA of *n*_H_ showed a significant MHC-TnT interaction effect (*P* < 0.05), suggesting that the TnT_R134W_-mediated impact on *n*_H_ was dissimilar in α- and β-MHC fiber bundles. *Post-hoc* analysis revealed that TnT_R134W_ showed no effect (*P* = 0.30; Figure 3D) on *n*_H_ in α-MHC fiber bundles, but it significantly decreased *n*_H_ by 26% (*P* < 0.001; Figure 3D) in β-MHC fiber bundles. These observations suggest that TnT_R134W_ does not affect myofilament cooperativity in the presence of α-MHC, but attenuates myofilament cooperativity in the presence of β-MHC. Collectively, these observations demonstrate that α- and β-MHC isoforms differently modulate the TnT_R134W_-mediated impact on thin filaments at submaximal Ca^2+^ activation.

### TnT_R134W_-mediated impact on XB detachment kinetics in α- and β-MHC fiber bundles

To determine whether TnT_R134W_ affected the XB detachment rate (*g*) in an MHC-dependent manner, we assessed tension cost and *c*. Tension cost was estimated as the slope of the linear relationship between tension and ATPase data at various pCa (de Tombe and Stienen, 1995; Stienen et al., 1995; Ford and Chandra, 2013). Within the context of a two-state XB model (Huxley, 1957), the ratio of steady-state ATPase activity (*fg*/*(f* + *g*)) and tension (*f*/*(f* + *g*)) is proportional to *g*; thus, tension cost is an approximate measure of *g*. *c*, which is the rate constant of the immediate force decay, following a sudden change in ML (Ford et al., 2010), is also a measure of *g* because it is positively correlated to tension cost (Campbell et al., 2004).

**Figure 4:**
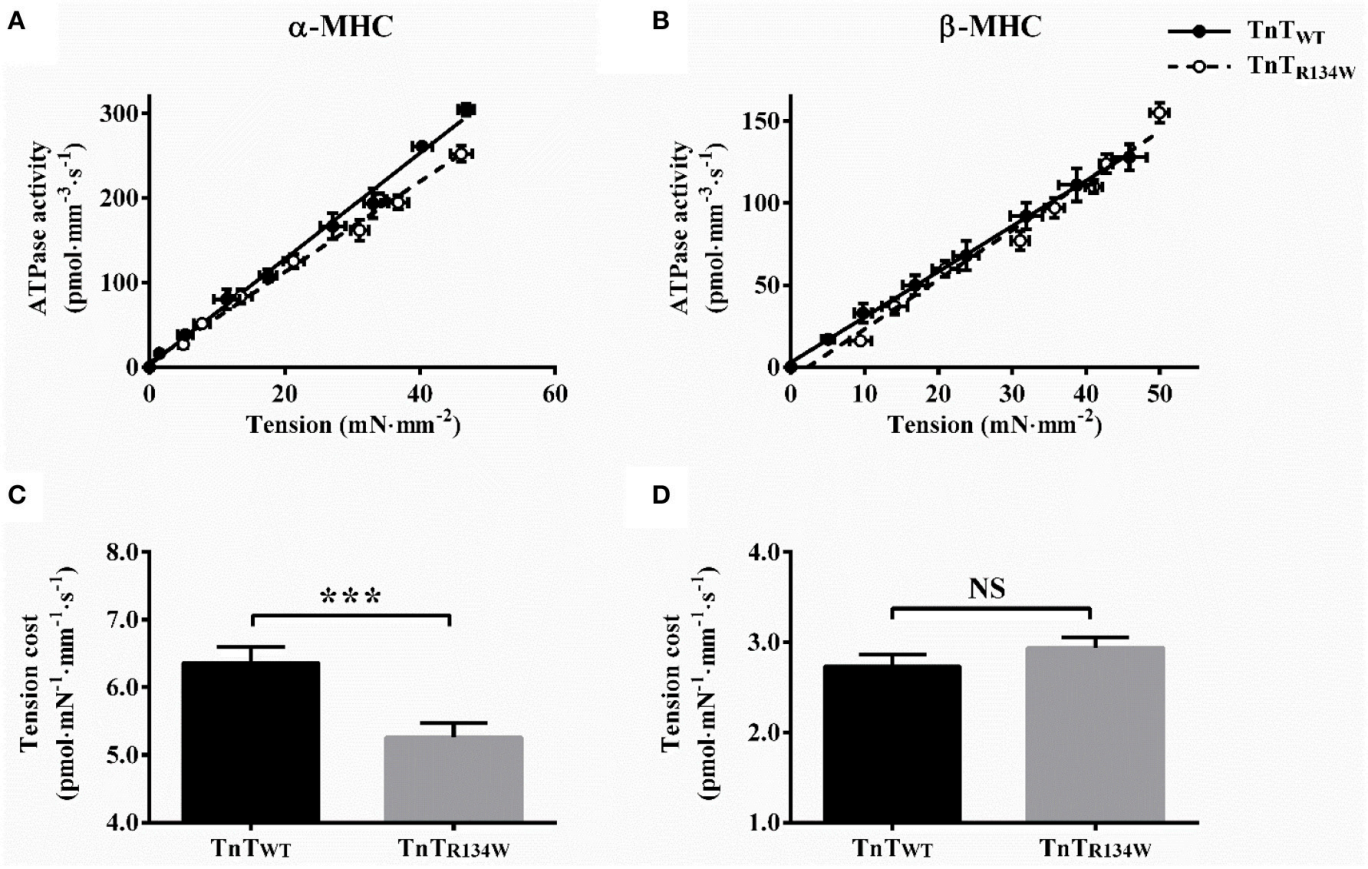
**Effect of TnT_**R134W**_ on tension cost in α- and β-MHC fiber bundles**. Simultaneous measurements of steady-state tension and ATPase activity were made in muscle fiber bundles at various pCa, as described in Materials and Methods. Tension cost was estimated as the slope of the linear regression fit to the tension-ATPase plot. TnT_R134W_-mediated impact on tension-ATPase relation in **(A)** α-MHC and **(B)** β-MHC fiber bundles. Traces connecting the data (empty or filled circles) are the linear regression fits. TnT_R134W_-mediated impact on tension cost in **(C)** α-MHC and **(D)** β-MHC fiber bundles. Statistical differences were analyzed by two-way ANOVA and subsequent *post-hoc t*-tests using Fishers LSD method. ^***^P < 0.001 indicate a significant result compared to TnT_WT_ (NS, not significant). The number of fiber bundles measured is as follows: 13 for α-MHC+TnT_WT_, 12 for α-MHC+TnT_R134W_, 14 for β-MHC+TnT_WT_, and 14 for β-MHC+TnT_R134W_. Data are presented as mean ± SE.

**Figure 5:**
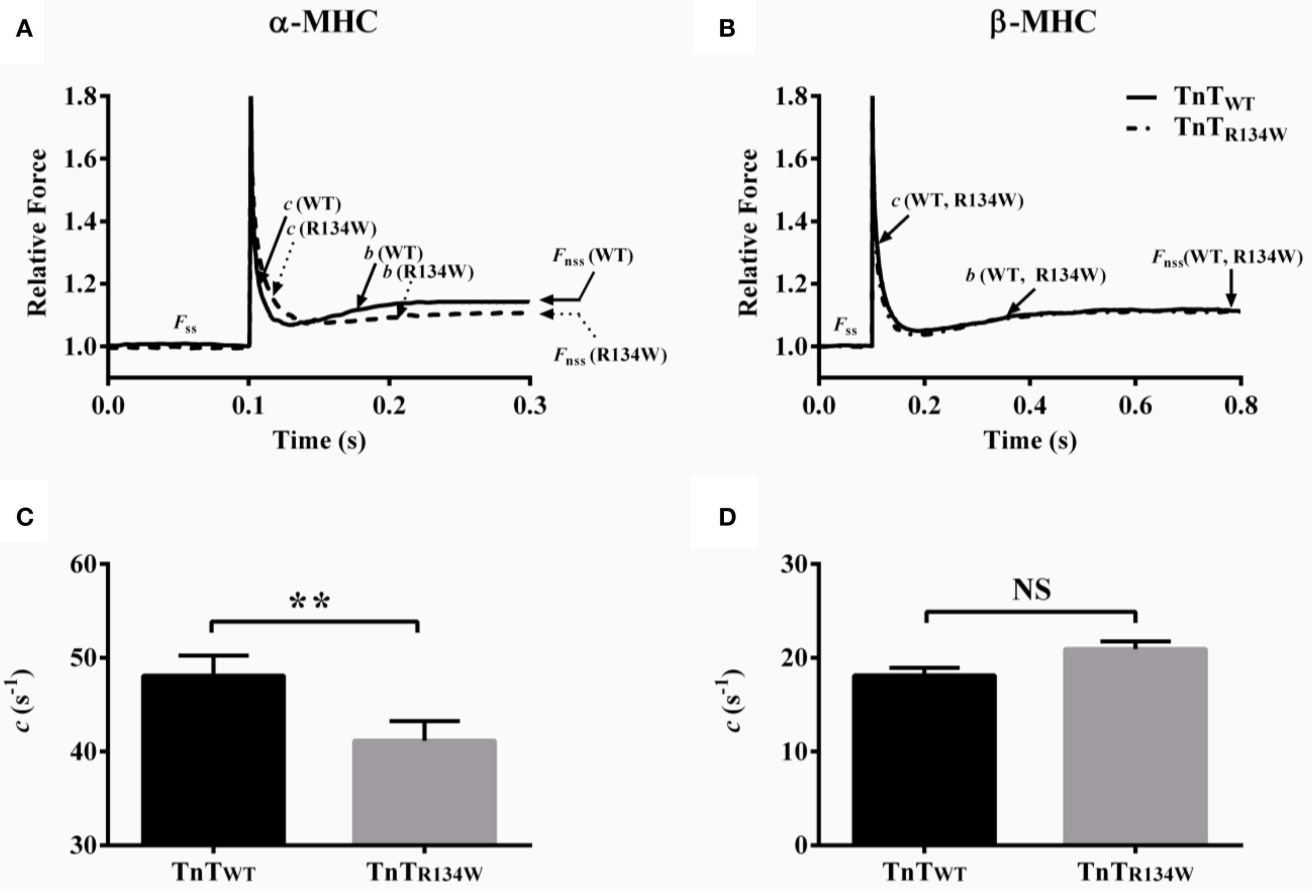
**Effect of TnT_**R134W**_ on ***c*** in α- and β-MHC fiber bundles**. TnT_R134W_-mediated impact on force response to a 2% stretch in muscle length (ML) in **(A)** α-MHC and **(B)** β-MHC fiber bundles. Force data were normalized by the isometric steady-state value, *F*_ss_, prior to stretch. *c* describes the rate of force decay to a minimum force point (nadir), *b* governs the rate of delayed force rise following an increase in ML, and *F*_nss_ represents the new steady-state force following an increase in ML. TnT_R134W_-mediated impact on *c* in **(C)** α-MHC and **(D)** β-MHC fiber bundles. Statistical differences were analyzed by two-way ANOVA and subsequent *post-hoc t*-tests using Fishers LSD method. ^**^P < 0.01 indicate a significant result compared to TnT_WT_ (NS, not significant). The number of fiber bundles measured is as follows: 13 for α-MHC+TnT_WT_, 12 for α-MHC+TnT_R134W_, 14 for β-MHC+TnT_WT_, and 14 for β-MHC+TnT_R134W_. Data are presented as mean ± SE.

A comparison showed that TnT_R134W_ induced a downward shift in the tension-ATPase plot in α-MHC fiber bundles (Figure 4A), which suggested a decrease in the slope of this relationship. On the other hand, TnT_R134W_ showed no effect on the tension-ATPase plot in β-MHC fiber bundles (Figure 4B). These disimilar effects of TnT_R134W_ on tension cost in α- and β-MHC fiber bundles gave rise to a significant MHC-TnT interaction effect (*P* < 0.001). *Post-hoc t*-tests showed that TnT_R134W_ significantly decreased tension cost by 17% (*P* < 0.001; Figure 4C) in α-MHC fiber bundles, while it showed no effect (*P* = 0.39; Figure 4D) in β-MHC fiber bundles. Observed effects in tension cost were also validated by our findings in *c*. A comparison of force responses to 2% stretch showed that TnT_R134W_ induced a rightward shift in the immediate force decay phase in α-MHC fiber bundles (Figure 5A), which suggested a slower *c*. However, TnT_R134W_ showed no effect on the immediate force response in β-MHC fiber bundles (Figure 5B). Two-way ANOVA showed a significant MHC-TnT interaction effect (*P* < 0.01) on *c*, which suggested that the effect of TnT_R134W_ on *c* was different in α- and β-MHC fiber bundles. *Post-hoc t*-tests showed that TnT_R134W_ significantly decreased *c* by 15% (*P* < 0.01; Figure 5C) in α-MHC fiber bundles, while it showed no effect (*P* = 0.21; Figure 5D) in β-MHC fiber bundles. Similar effects in tension cost and *c* suggest that TnT_R134W_-induced changes in thin filaments interact differently with those induced by α- and β-MHC isoforms to differently modulate the effect on *g*.

### TnT_R134W_-mediated impact on XB turnover rate in α- and β-MHC fiber bundles

To determine whether TnT_R134W_ differently altered XB turnover rate in α- and β-MHC fiber bundles, we assessed two independent rate parameters, *k*_tr_ and *b*. While *k*_tr_ represents the rate of force redevelopment following a large release-restretch length maneuver (Brenner and Eisenberg, 1986), *b* describes the rate of delayed force rise following a sudden stretch in ML (Ford et al., 2010). Both *k*_tr_ and *b* have been previously shown to be approximate measures of XB turnover rate (Ford et al., 2010; Gollapudi et al., 2013, 2015; Chandra et al., 2015).

**Figure 6:**
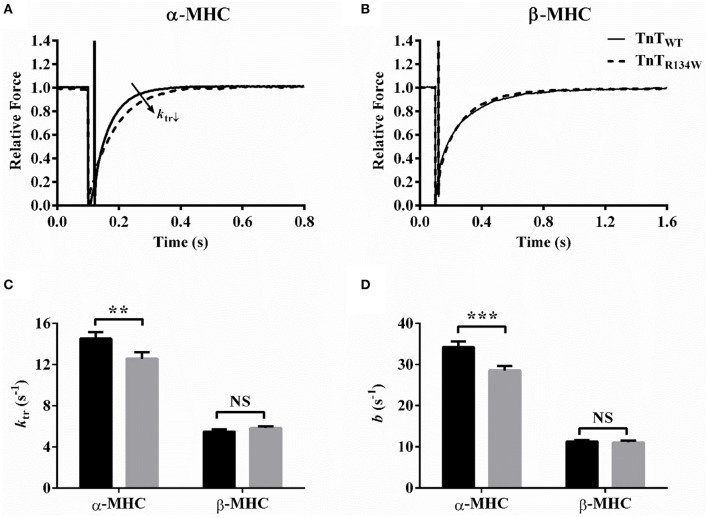
**Effect of TnT_**R134W**_ on ***k***_**tr**_ and ***b*** in α- and β-MHC fiber bundles**. *k*_tr_ was estimated by fitting a mono-exponential function to the rising phase of the force response following a large release-restretch length maneuver (Brenner and Eisenberg, 1986). *b* was estimated by fitting the NLRD model to a family of force responses to various amplitude ML perturbations (Ford et al., 2010). TnT_R134W_-mediated effect on the force response to a large release-restretch maneuver in **(A)** α-MHC and **(B)** β-MHC fiber bundles. Force data were normalized by the isometric steady-state value following the length perturbation. TnT_R134W_-mediated effect on **(C)** *k*_tr_ and **(D)** *b* in α- and β-MHC fiber bundles. Statistical differences were analyzed by two-way ANOVA and subsequent *post-hoc t*-tests using Fishers LSD method. ^**^P < 0.01 and ^***^P < 0.001 indicate significant results compared to TnT_WT_ (NS, not significant). The number of fiber bundles measured is as follows: 13 for α-MHC+TnT_WT_, 12 for α-MHC+TnT_R134W_, 14 for β-MHC+TnT_WT_, and 14 for β-MHC+TnT_R134W_. Data are presented as mean ± SE.

A representative comparison of force responses to a large release-restretch length meneuver showed that, in α-MHC fiber bundles (Figure 6A), TnT_R134W_ induced a rightward shift in the rising force phase, which suggested a slower rate of force rise. On the contrary, TnT_R134W_ showed no effect on the force response in β-MHC fiber bundles (Figure 6B). Therefore, two-way ANOVA showed a significant MHC-TnT interaction effect on *k*_tr_ (*P* < 0.05), which suggested that the effects of TnT_R134W_ on *k*_tr_ against α- and β-MHC were dissimilar. *Post-hoc* analysis confirmed that TnT_R134W_ attenuated *k*_tr_ by 14% (*P* < 0.01; Figure 6C) in α-MHC fiber bundles, while it showed no effect (*P* = 0.57; Figure 6C) in β-MHC fiber bundles. Our analysis of *b* also revealed similar findings. A comparison of force responses to a 2% stretch showed that TnT_R134W_ induced a rightward shift in the delayed force rise phase in α-MHC fiber bundles (Figure 5A), which suggested attenuation of *b*. However, TnT_R134W_ displayed no effect on the delayed force rise phase in β-MHC fiber bundles (Figure 5B). These differential effects of TnT_R134W_ on *b* in α- and β-MHC fiber bundles gave rise to a significant MHC-TnT interaction effect (*P* < 0.01). *Post-hoc* analysis confirmed that TnT_R134W_ attenuated *b* by 17% (*P* < 0.001; Figure 6D) in α-MHC fiber bundles, while it showed no effect (*P* = 0.86; Figure 6D) in β-MHC fiber bundles. Therefore, similar effects in *k*_tr_ and *b* demonstrate that the XB turnover rate is attenuated by TnT_R134W_ only in the presence of α-MHC.

### TnT_R134W_-mediated impact on *E*_R_ in α- and β-MHC fiber bundles

**Figure 7:**
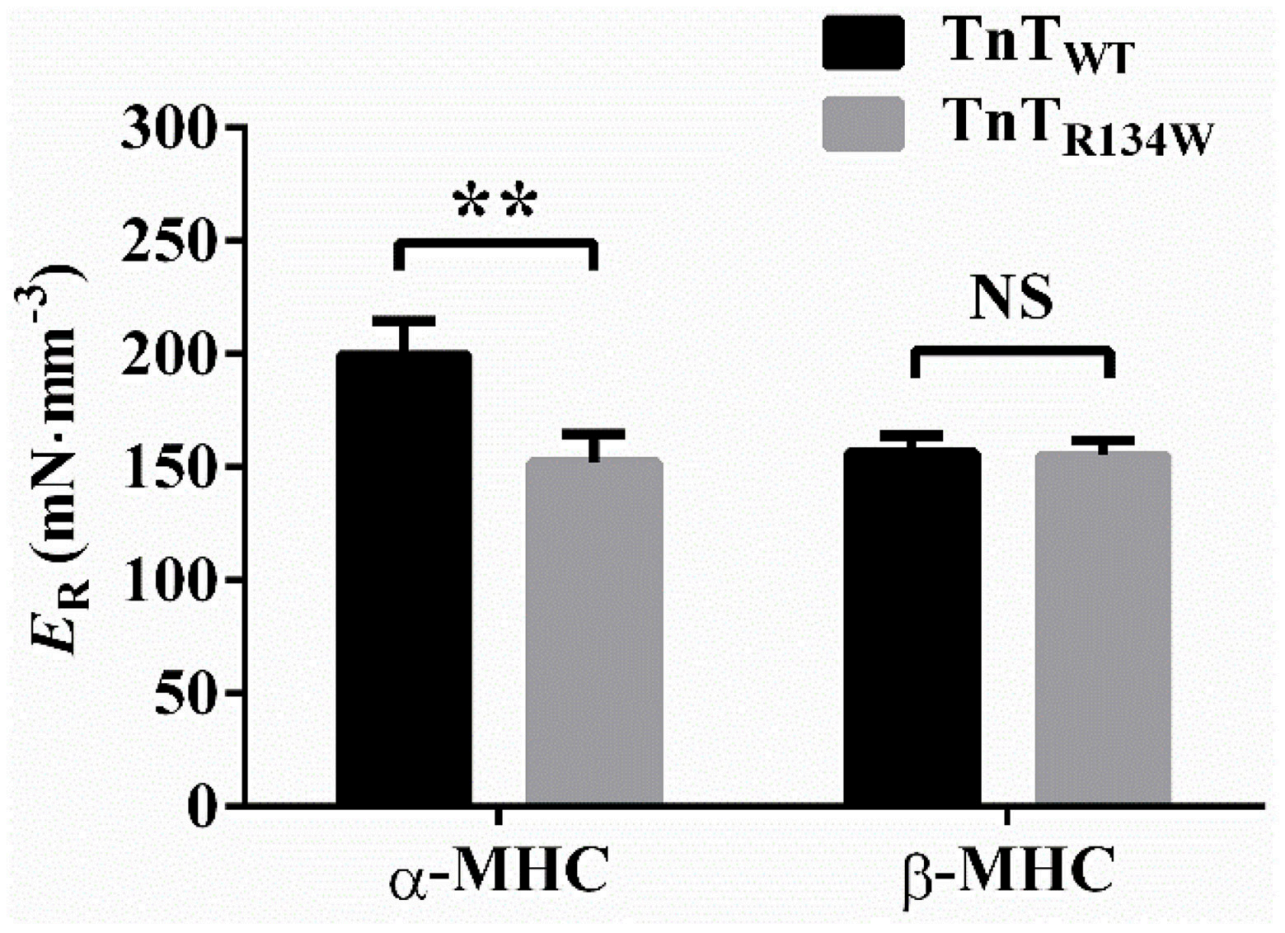
**Effect of TnT_**R134W**_ on ***E***_**R**_ in α- and β-MHC fiber bundles**. *E*_R_ was estimated as the slope of a linear relationship between (*F*_nss_ − *F*_ss_) elicited by muscle fiber bundles to imposed ML changes, ΔL (see Figures 5A,B for details on *F*_nss_ and *F*_ss_). Statistical differences were analyzed by two-way ANOVA and subsequent *post-hoc t*-tests using Fishers LSD method. ^**^P < 0.01 indicate a significant result compared to TnT_WT_ fibers (NS, not significant). The number of fiber bundles measured is as follows: 13 for α-MHC+TnT_WT_, 12 for α-MHC+TnT_R134W_, 14 for β-MHC+TnT_WT_, and 14 for β-MHC+TnT_R134W_. Data are presented as mean ± SE.

To investigate whether TnT_R134W_ differentially altered the magnitude of stretch activation in α- and β-MHC fiber bundles, we assessed estimates of *E*_R_ at maximal Ca^2+^ activation (pCa 4.3). *E*_R_ represents the magnitude of muscle length-mediated recruitment of new force-bearing XBs (*E*_R_) and is equivalent to the magnitude of stretch activation (Campbell and Chandra, 2006; Stelzer et al., 2006b, 2007; Ford et al., 2010). *E*_R_ is derived as the slope of the linear regression between (*F*_nss_ − *F*_ss_) and Δ*L* (see Figures 5A,B), where *F*_nss_ is the force corresponding to the new-steady state attained after the change in ML, *F*_ss_ is the steady-state isometric force prior to the change in ML, and Δ*L* is the imposed ML change. Thus, *E*_R_ increases when *F*_nss_ increases and vice versa. Comparison of force responses to a 2% stretch showed that TnT_R134W_ attenuated *F*_nss_ in α-MHC fiber bundles (Figure 5A), which suggested a decrease in *E*_R_. On the other hand, TnT_R134W_ showed no effect on *F*_nss_ in β-MHC fiber bundles (Figure 5B). These different effects of TnT_R134W_ on *E*_R_ in α- and β-MHC fiber bundles gave rise to a significant MHC-TnT interaction effect (*P* < 0.05). *Post-hoc* analysis showed that TnT_R134W_ significantly decreased *E*_R_ by 24% (*P* < 0.01; Figure 7) in α-MHC fiber bundles, while it showed no effect (*P* = 0.92; Figure 7) in β-MHC fiber bundles. These observations demonstrate that the magnitude of stretch activation mediated by TnT_R134W_ is differently altered by α- and β-MHC.

## Discussion

The severity of DCM phenotypes in humans varies so widely that a commonly attributed change in a steady-state contractile parameter, such as a modest decrease in myofilament Ca^2+^ sensitivity, precludes us from explaining disparate cardiac phenotypes. Because dynamic aspects of cardiac contraction dominate under conditions in which the heart muscle normally operates, dynamic contractile indices may provide more meaningful clues to link disparate phenotypes to different mutations. Our extensive steady-state and dynamic contractile data demonstrate that alterations in contractile dynamics (both rate and magnitude), in addition to the differential impact of α- and β-MHC on myofilament Ca^2+^ sensitivity, allow us to expand our view on how some mutations in TnT affect heart function and cardiac phenotypes.

### TnT_R134W_ attenuates myofilament Ca^2+^ sensitivity to a greater extent in α-MHC than in β-MHC fiber bundles

A greater magnitude of decrease in myofilament Ca^2+^ sensitivity in α-MHC+TnT_R134W_ than in β-MHC+TnT_R134W_ fiber bundles (Figure 3C) raises two questions: (1) how does TnT_R134W_ attenuate thin filament activation?; and (2) why is the effect of TnT_R134W_ on thin filaments minimized in the presence of β-MHC? Previous studies have associated residues 112–136 of human TnT in the strong interaction of CR of TnT with Tm at the Tm-Tm overlap junction (Hinkle and Tobacman, 2003). This CR-Tm interaction acts as a gateway not only for the activation of regulatory units (RU; Tn-Tm complex) but also for cooperative interactions between near-neighbor RUs and between near-neighbor RUs and XBs (Schaertl et al., 1995; Razumova et al., 2000; Tobacman et al., 2002; Moss et al., 2004). There is evidence to suggest that the R131W mutation in TnT decreases RU activation by increasing the rate of dissociation of Ca^2+^ from Tn (Liu et al., 2012). While such attenuation of RU activation may primarily involve altered CR-Tm interaction, another study suggests that the R131W-induced structural changes in the CR may also spread to the T2 region of TnT to modify Ca^2+^-sensitive interactions between TnT and TnI/TnC (Mogensen et al., 2004). Based on these findings, we posit that TnT_R134W_ alters allosteric/cooperative mechanisms that underlie RU activation. At submaximal [Ca^2+^], such actions of TnT_R134W_ increase the amount of Ca^2+^ required to attain the magnitude of RU activation that is normally observed in TnT_WT_, leading to attenuation of pCa_50_ in both α- and β-MHC fiber bundles. However, the magnitude of the impact on pCa_50_ is lower in β-MHC+TnT_R134W_ than in α-MHC+TnT_R134W_ fiber bundles, suggesting that β-MHC partially counters the effect of TnT_R134W_ on RU activation. Given that enhanced XB-RU, but not XB-XB, cooperativity increases pCa_50_ (Razumova et al., 2000), the ability of β-MHC to counter the influence of TnT_R134W_ on pCa_50_ appears to arise from greater XB-RU cooperativity.

How differences in XB cycling kinetics permit α- and β-MHC (Rundell et al., 2005; Stelzer et al., 2007; Ford and Chandra, 2013) to differently modify XB-RU cooperativity may be gleaned by considering the initial conditions of thin filaments. At submaximal [Ca^2+^]_free_, the TnT_R134W_-induced attenuation of RU activation leaves behind a larger than normal pool of RUs in the *off* state, thereby increasing the scope for strong XBs to cooperatively influence XB-RU interactions. Therefore, the slow cycling β-MHC may exert a positive effect on thin filaments by amplifying XB-RU cooperativity. This enhanced XB-RU cooperativity by β-MHC is expected to facilitate the transition of RUs from the *off* to the *on* state, thereby resulting in an increase in RU activation and a subsequent increase in the number of force-bearing XBs. Indeed, tension is augmented at submaximal activation in β-MHC+TnT_R134W_ fiber bundles when compared to α-MHC+TnT_R134W_ fiber bundles. This explains why β-MHC is able to partially counter the negative influence of TnT_R134W_ on pCa_50_. Such β-MHC-mediated increase in XB-RU cooperativity may have exhausted the pool of RUs from which RU-RU cooperativity could recruit (Razumova et al., 2000), thereby decreasing the contributions of RU-RU cooperativity to *n*_H_ in β-MHC+TnT_R134W_ fiber bundles. Because RU-RU cooperativity has the greatest influence on *n*_H_ (Razumova et al., 2000), a decrease in RU-RU cooperativity may likely explain the decrease in *n*_H_ in β-MHC+TnT_R134W_ fiber bundles.

### β-MHC neutralizes the attenuating effect of TnT_R134W_ on XB turnover rate and XB detachment rate

Our observations on two contractile rate parameters, *k*_tr_ and *b* (Figure 6), confirm that TnT_R134W_ attenuates XB turnover rate in α-MHC fiber bundles, but shows no effect in β-MHC fiber bundles. In our previous studies (Campbell, 1997; Campbell et al., 2004; Ford et al., 2010; Gollapudi and Chandra, 2012), we have shown that attenuation of *b* may be brought about by the following: (1) attenuation of RU *on*/*off* kinetics; (2) attenuation of XB cycling kinetics, *f* and *g*; (3) augmentation of XB-based cooperativity; or (4) a combinatorial effect of 1, 2, and 3. However, unaltered maximal tension and *E*_D_ in α-MHC+TnT_R134W_ fiber bundles suggest that the impact of TnT_R134W_ on RU activation is minimized. Under these conditions, the available pool of RUs and XBs from which XB-based cooperativity may recruit is expected to be similar in both α-MHC+TnT_WT_ and α-MHC+TnT_R134W_ fiber bundles. Thus, it is unlikely that enhanced XB-based cooperativity is responsible for slower *b* in α-MHC+TnT_R134W_ fiber bundles. Therefore, our observations suggest that attenuation of *b* may result from the slowing effect of TnT_R134W_ on XB cycling kinetics. Because *k*_tr_ = *f* + *g*, as per the two-state XB model (Huxley, 1957; Brenner, 1988; de Tombe and Stienen, 2007), a significant attenuation of *k*_tr_ in α-MHC+TnT_R134W_ fiber bundles also substantiates our assertion that attenuation of *b* is due to a slowing effect on *f* and/or *g*.

Evidence to substantiate that attenuation of *g* may underlie the slowed XB turnover rate (*k*_tr_ and *b*; Figure 6) in α-MHC+TnT_R134W_ fiber bundles comes from our observations on tension cost (Figure 4) and *c* (Figure 5). Furthermore, other observations suggest that the attenuation of XB turnover rate in α-MHC+TnT_R134W_ fiber bundles may also involve a slowing effect on *f*; this is because steady-state isometric force is proportional to *f*/(*f* + *g*) (Huxley, 1957). Therefore, a decrease in *g* alone should increase force produced in α-MHC+TnT_R134W_ fiber bundles. However, both maximal tension and *E*_D_ are unaltered in α-MHC+TnT_R134W_ fiber bundles, which suggests that TnT_R134W_ does not impact *f*/(*f* + *g*) in the presence of α-MHC at maximal activation. Thus, this conjectural evidence may indicate that *f* decreases in proportion to *g* in α-MHC+TnT_R134W_ fiber bundles. In contrast, a lack of effect on *g* in β-MHC+TnT_R134W_ fiber bundles (Figures 4, 5) suggests that β-MHC negates the attenuating effect of TnT_R134W_ on *g*. Our observation on *g*, in conjunction with unaltered *k*_tr_, *b*, and maximal tension in β-MHC+TnT_R134W_ fiber bundles, suggests that TnT_R134W_ does not alter *f* in the presence of β-MHC. These observations demonstrate that the interplay between the TnT- and MHC-mediated effects on the thin filament modulate XB cycling kinetics.

### β-MHC neutralizes the attenuating effect of TnT_R134W_ on the magnitude of stretch activation

Another notable finding from our study is that TnT_R134W_ attenuates the magnitude of stretch activation (*E*_R_) in α-MHC but shows no effect in β-MHC fiber bundles (Figure 7). The magnitude of *E*_R_ is dependent on the ML-related XB recruitment mechanisms that operate within thin filaments. For example, XB-based (XB-RU/XB-XB) cooperativity, which is mediated through thin filaments, strongly influences *E*_R_ such that a decrease in XB-based cooperativity decreases *E*_R_ and *vice versa* (Campbell et al., 2004; Campbell and Chandra, 2006; Stelzer et al., 2006b). Our data suggest that different outcomes on *E*_R_ in α-MHC+TnT_R134W_ and β-MHC+TnT_R134W_ fiber bundles may be closely linked to differential effects on XB-RU cooperativity. To clarify, a decrease in XB-RU cooperativity may be responsible for the attenuation of *E*_R_ in TnT_R134W_+α-MHC fiber bundles, while unaltered XB-RU cooperativity explains why *E*_R_ is unaffected in β-MHC+TnT_R134W_ fiber bundles. One source of this difference between α- and β-MHC fiber bundles may be related to our earlier assertion that the negative effect of TnT_R134W_ on RU activation remains more prominent in α-MHC than in β-MHC fiber bundles. Therefore, *E*_R_ is attenuated in α- but not in β-MHC fiber bundles.

### Implications of our findings for heart function in mice and humans

TnT_R134W_ attenuated XB turnover and detachment rates in α-MHC fiber bundles, but not in β-MHC fiber bundles. When extrapolated to the whole heart level, these observations suggest slower rise and slower fall of ventricular pressure in α-MHC containing hearts but not in β-MHC containing hearts. In addition, slower rates of XB turnover and detachment may also slow dynamics of ejection in α-MHC-expressing hearts. Previous studies also implicate mechanisms such as stretch activation in maintaining the ventricular force output during the late phase of ejection (Stelzer et al., 2006a,c). Thus, attenuation of *E*_R_ in α-MHC+TnT_R134W_ fiber bundles, in conjunction with slowed XB turnover and detachment rates, suggests that the ejection phase may be prematurely terminated in α-MHC-expressing hearts. Inferences drawn from dynamic studies demonstrate that the magnitude of cardiac contractile impairment, induced by TnT_R134W_, differ significantly in α- and β-MHC expressing fiber bundles. The effect on pCa_50_ also shows that the severity of contractile deficits induced by TnT_R134W_ is different in α- and β-MHC fiber bundles; for instance, TnT_R134W_ decreases pCa_50_ to a greater extent in α-MHC fiber bundles (Figure 3C). Although the attenuation of pCa_50_ alone may suggest DCM in both α- and β-MHC background, the severity of cardiac phenotype is expected to be greater in mouse hearts because various indices of contractile dynamics were attenuated only in α-MHC fiber bundles.

## Author contributions

Contribution of SG: Conception and design, acquisition of data, analysis and interpretation of data, drafting and revising the manuscript. Contribution of MC: Conception and design, interpretation of data, drafting and revising the manuscript.

## Funding

This work was supported, in part, by National Institutes of Health Grant No. HL-075643 (to MC) and a Poncin grant supported by the Autzen foundation.

### Conflict of interest statement

The authors declare that the research was conducted in the absence of any commercial or financial relationships that could be construed as a potential conflict of interest.

## Abbreviations

ANOVA: analysis of variance
CR: central region
DCM: dilated cardiomyopathy
HR: high relaxing
LSD: least significant differences
MHC: myosin heavy chain
ML: muscle length
NLRD: nonlinear recruitment distortion
NTG: non-transgenic
RU: regulatory unit
SL: sarcomere length
TG: transgenic
Tm: tropomyosin
Tn: troponin
TnC: troponin C
TnI: troponin I
TnT: troponin T
WT: wild-type
XB: crossbridge.
